# Modeling Uniaxial Bond Stress–Slip Behavior of Reinforcing Bars Embedded in Concrete with Different Strengths

**DOI:** 10.3390/ma14040783

**Published:** 2021-02-07

**Authors:** Chao-Wei Tang

**Affiliations:** 1 Department of Civil Engineering and Geomatics, Cheng Shiu University, No. 840, Chengching Rd., Niaosong District, Kaohsiung 83347, Taiwan; tangcw@gcloud.csu.edu.tw; Tel.: +886-7-735-8800; 2 Center for Environmental Toxin and Emerging-Contaminant Research, Cheng Shiu University, No. 840, Chengcing Rd., Niaosong District, Kaohsiung 83347, Taiwan; 3 Super Micro Mass Research and Technology Center, Cheng Shiu University, No. 840, Chengching Rd., Niaosong District, Kaohsiung 83347, Taiwan

**Keywords:** reinforcing bar, bond stress, slip, uniaxial tensile test

## Abstract

This paper aims to study the uniaxial bond stress–slip characteristics of reinforcing bars embedded in concrete with different strengths. Tests were conducted on tension–pull specimens that had a cross-sectional dimension with a reinforcing bar embedded in the center section. The experimental variable was the concrete compressive strength (20, 40, and 60 MPa). The test results show that in the specimen subjected to any fixed load, the maximum value of the concrete strain occurred around the central position, and its value increased as the compressive strength of the concrete increased. Depending on the embedded position of the steel bars, the bond stress–slip relationship was also different. In addition, the analytical results indicate that the proposed bond stress–slip constitutive relationship is very accurate in describing the true bond stress–slip relationship.

## 1. Introduction

Reinforced concrete (RC) is a composite material that has been widely used in civil engineering due to its advantages such as durability, fire resistance, and cost effectiveness [1]. The structural performance of RC members mainly depends on a sufficient bond between the reinforcing bars and the surrounding concrete [2,3]. For RC members, the characteristic of bond slip is the difference in material strain at a certain point along the reinforcing bar. Because the bond–slip relationship at the interface between steel and concrete is very complex and involves multiple variables, actual structural analysis usually ignores the effect of bond–slip on the overall mechanical properties of the RC structures. In other words, it is assumed that the steel and concrete are completely bonded, so the relative slip between the two is ignored, resulting in a large deviation from the analysis results.

So far, many scholars have established corresponding bond–slip constitutive relations and models based on a large number of experimental studies under comprehensive consideration of various factors affecting the bond performance of RC members. On this basis, some scholars combined experimental research and theoretical analysis to establish different reinforced concrete bond–slip theoretical models and numerical analogy methods. By bringing the bond–slip constitutive relationship into the structural analysis to consider the bond–slip effect, the analogy accuracy of the structural response is improved. Regarding the issue of the bond–slip between steel and concrete, this study reviewed the relevant literature from three aspects: bond–slip mechanism, experimental research, and theoretical model and numerical simulation as shown in the following paragraphs.

For deformed steel bars, the force transmission from the steel bars to the surrounding concrete occurs through the following mechanisms: chemical adhesion, friction resistance, and mechanical interlock [3]. The chemical adhesion between the steel bars and the concrete has only a slight influence. The frictional forces are caused by the roughness of the interface, forces transverse to the bar surface, and relative slip between the bar and the surrounding concrete. The mechanical interlock due to the surface protrusions or ribs provided in deformed steel bars is the most critical mechanism. In structural design, it is very impractical to determine the bond strength by measuring the three component stresses of the bond stress from a microscopic point of view. In order to simplify the complexity of calculation, many scholars have put forward the concept of average bond stress to embody the bond strength between steel bars and concrete. For example, Filippou et al. [4] supposed that the bond stress was uniformly distributed along the embedment length. Therefore, based on the force equilibrium between the loads on the bar and the available bond resistance, the following equation could be established:

(1) $$\Delta T=\frac{\pi d_{b}^{2}}{4}d\sigma_{s}=\tau \pi d_{b}dx$$

where τ is the bond stress and $\sigma_{s}$ is the reinforcing bar stress, $d_{b}$ is the diameter of the reinforcing bar, and dx or Δx is the embedment length of the reinforcing bar. Then, the average bond stress could be expressed as follow:

(2) $$\tau \left(x\right)=\frac{\Delta T}{\pi d_{b}\Delta x}=\frac{d_{b}}{4}\frac{d\sigma_{s}}{dx}$$

There are two commonly used test methods for the experimental study of the bond–slip relationship of RC components. One is the pull-out test, and the other is the beam test (axial tensile test) [5,6]. In most pull-out tests, the embedded length of the reinforcing bar is set to be short (usually no more than five times the diameter of the steel bar), the reinforcing bar more or less maintained in the elastic stage, and the bond stress is approximately constant [7]. When the embedded length is long, on the one hand, the steel bar undergoes significant strain due to greater stress, and the steel bar slips due to elongation under strain penetration [8,9]. On the other hand, under lateral confinement, once the tensile yield occurs, the transverse steel shrinks due to the Poisson effect, which affects the development of radial compressive stress. Based on the friction mechanism, the bond strength is reduced [10]. In addition, the yield of the steel bar also affects the geometry of the rib and further weakens the bond strength. Therefore, considering the effect of the steel bar yield and strain penetration, it is worth establishing a bond–slip relationship suitable for long anchorage. Shima et al. [11] studied the bond performance of steel bars after yielding by conducting pull-out tests on reinforced concrete specimens with an anchor length of $50d_{b}$. The experimental study found that the steel bar strain had a significant effect on the bond–slip relationship. In the elastic stage, the strain curve was very smooth, but when the steel bar yielded and began to enter the hardening stage, the bond stress declined sharply. To further study the bond–slip relationship under strain penetration, Liang and Sritharan [12] designed a total of five sets of specimens with an embedded length of $48d_{b}$ and carried out pull-out tests using monotonic loading and cyclic loading. A corresponding analysis model was also established.

With the development of concrete technology, concretes with various properties have been successively applied to actual projects. In view of this, scholars have also explored the bond–slip constitutive relationship of these concretes that are different from the traditional concrete composition. Cui et al. [13] used a standard beam-end pull-out test to conduct an experimental study on the bond stress of steel bars in reinforced geopolymer concrete (GPC) structures. The results show that the GPC specimens had high bond stiffness. Compared with ordinary Portland cement concrete, GPC showed that it can withstand greater tensile loads at the same relative slip value. Huang et al. [14] studied the bond strength between deformed steel bars and steel–polypropylene hybrid fiber reinforced concrete (HFRC). Through a series of monotonic/cyclic pull-out tests, the benefits of hybrid fibers were evaluated. Moreover, an analytical model was proposed to estimate the ultimate bond strength, which was well verified by other independent experimental results.

On the other hand, due to the increasing awareness of environmental protection, the composition of concrete has been replaced by renewable resources. Romanazzi et al. [15] studied the bond–slip behavior between rubberized concrete (RuC) and deformed steel bars. It was observed that when the percentage of rubber particles to replace natural fine aggregate exceeded 12%, the bond strength decreased (up to 20% relative to the reference mixture). Gao et al. [16] studied the bond performance between deformed steel bars and steel–polypropylene hybrid fiber reinforced recycled aggregate concrete (HFRAC). The results clearly demonstrated that the steel–polypropylene hybrid fiber could synergistically increase the bond strength between the steel bar and HFRAC. Rockson et al. [17] studied the bond strength between steel bars and structural concrete using commercially produced recycled coarse and fine aggregates. The results showed that when using recycled concrete to design structures, the current design codes and empirical formulas found in the literature were conservative.

**Figure 1:**
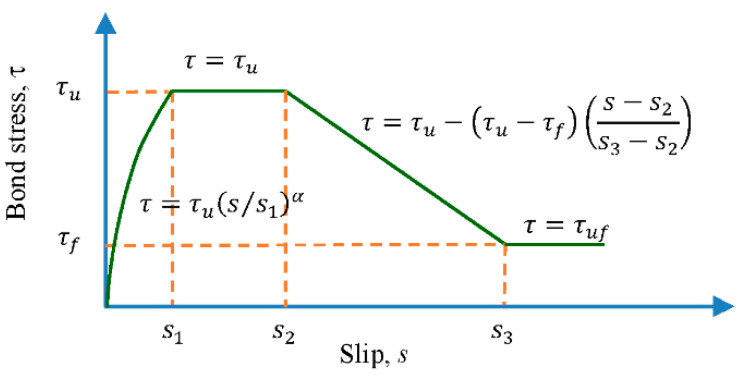
Analytical bond stress–slip relationship (CEB-FIP Model Code 2010).

The literature shows that the bond stress–slip relationship depends on many factors or conditions, which can be roughly summarized into four categories: concrete properties; reinforcement properties; stress state; and loading type [18]. In view of this, in the past few decades, scholars have proposed various bond stress–slip relationships and corresponding bond models. Eligehausen et al. [7] proposed a bond model in 1983, claiming that the bond strength increased with the increase in concrete strength and could be regarded as a function of the square root of the concrete compressive strength. Moreover, in 1983, Filippou et al. [4] established an analytical model to describe the hysteresis performance of reinforced concrete beam–column joints. Therefore, this model was collectively referred to as the Eligehausen–Filippou model. Then, the CEB-FIP model code 2010 [19] adopted this model. Taking the nonlinear bond stress–slip relationship of the CEB-FIP Model Code 2010 as an example, if it is a failure of the pullout, the bond stress (τ) between the concrete and steel bar as a function of the relative displacement (s) can be calculated by the following equations:

(3) $$\tau =\tau_{u}{\left(s/s_{1}\right)}^{\alpha }\;\mathrm{for}\;0\leq s\leq s_{1}$$

(4) $$\tau =\tau_{u}\;\mathrm{for}s_{1}<s\leq s_{2}$$

(5) $$\tau =\tau_{u}-\left(\tau_{u}-\tau_{f}\right)\left(\frac{s-s_{2}}{s_{3}-s_{2}}\right)\;\mathrm{for}\;s_{2}<s\leq s_{3}\;$$

(6) $$\tau =\tau_{f}\;\mathrm{for}s_{3}<s$$

where $\tau_{u}$ is the peak bond stress; $\tau_{f}$ is the residual bond stress; *s* is the bond slip; and $s_{1}$, $s_{2},$ and $s_{3}$ are the slip at the start of peak bond stress, slip at the end of peak bond stress, and slip at the start of residual bond stress, respectively; *α* is a curve fitting parameter that must not be larger than one to be physically meaningful. At present, the local bond stress–slip relationship recommended by the CEB-FIP Model code 2010 is shown in Figure 1, and the corresponding parameters in the figure are shown in Table 1 [19,20,21]. The local bond stress–slip relationship suggested by Huang et al. [20] and Harajli et al. [21,22] is similar to that in Figure 1, and the corresponding parameters in the figure are shown in Table 1. On the other hand, regarding bond strength, the literature shows that different models give different prediction equations. Table 2 shows the maximum “local” bond strength observed in previous experimental tests, mainly from the pull-out tests. It can be seen from Table 2 that with the different types of concrete, there is obviously scatter in the values of maximum bond strength and its value ranges from 1.7$\sqrt{f_{c}^{\prime }}$ to 5.7$\sqrt{f_{c}^{\prime }}$.

Guizani et al. [31] produced 43 moderately anchored reinforced concrete specimens with an anchorage length of 5*d_b_*, and established their bond–slip constitutive relationship through pull-out tests. In order to consider the influence of the yield of steel bars, Marti et al. [32] assumed that the bond–slip relationship is ideal rigid-plasticity, and proposed a tensioned chord model, which can be applied to cracks, minimum reinforcement ratio, tension stiffness effect and rotation capacity and so on. Later, Lowes et al. [33] modified the bond–slip relationship based on a shorter anchorage length by introducing a reduction factor to consider the influence of the yield of the steel bar under long anchorage. Based on the aforementioned research, Fernández Ruiz et al. [34] made improvements, taking into account the influence of the shape of the steel bar. Two models (the square root model and the rigid-plastic model) were proposed, which could be used to describe the bond–slip relationship before and after yielding of the steel bar. Affected by the difference in boundary conditions, the empirical formulas and models derived from simple pull-out tests are not applicable to some parts of the structure and components. Therefore, Hong and Park [6] studied the bond stress–slip relationship of reinforced concrete members under axial tensile loads. An analytical model was proposed, which utilized the conventional bond stress–slip theory, the deformed bar characteristics, and the concrete cross-sectional area.

At high temperatures, the bond properties between steel and concrete will gradually decline, which will seriously affect the mechanical properties of the structure. Aslani and Samali [35] proposed a modified formula for the local bond stress–slip relationship of the CEB-FIP Model code 2010 to analyze the bond stress and relative slip between concrete and steel bars after exposure to high temperatures, as follows:

(7) $$\tau \left(T\right)=\tau_{u,T}{\left(s/s_{1}\right)}^{\alpha }\;\mathrm{for}\;\;0\leq s\leq s_{1}$$

(8) $$\tau \left(T\right)=\tau_{u,T}\;\mathrm{for}\;s_{1}<s\leq s_{2}$$

(9) $$\tau \left(T\right)=\tau_{u,T}-\left(\tau_{u,T}-\tau_{f,T}\right)\left(\frac{s-s_{2}}{s_{3}-s_{2}}\right)\;\mathrm{for}\;s_{2}<s\leq s_{3}$$

(10) $$\tau \left(T\right)=0.4\tau_{u,T}\;\mathrm{for}\;s_{3}<s$$

where τ(T)= the bond strength after exposure to high temperatures of *T* °C, $\tau_{u,T}$ = the ultimate bond strength after exposure to high temperatures of *T* °C; $\tau_{f,T}$ = the residual bond stress after exposure to high temperatures of *T* °C, *s* = the bond slip; $s_{1}$, $s_{2},$ and $s_{3}$ are the slip at the start of ultimate bond stress, slip at the end of ultimate bond stress, and slip at the start of residual bond stress, respectively; *α* is a curve fitting parameter that must not be larger than one.

In addition to the theoretical model of the bond–slip relationship, scholars have conducted extensive research on numerical simulation. The choice of modeling method can be direct or indirect [10]. The direct method is mainly to establish a refined finite element model and to consider the bond–slip phenomenon by inserting the corresponding bond element. For example, Lundgren and Gylltoft [36] developed a three-dimensional interface element that connects steel and concrete. In this model, the splitting stress due to bonding was included. In addition, the bond stress depended not only on the slip, but also on the radial deformation between the steel bar and the concrete. By inserting this interface element in the finite element software, a reasonable prediction of the split failure and bond stress loss of the steel bar after yielding could be realized. On the other hand, the indirect method starts from the influence of the bond–slip on the performance of structural members, by modifying the constitutive relationship of steel or concrete, or directly adding spring elements at the end of the member, giving the member extra flexibility, so as to indirectly consider the bond–slip effect. Dehestani and Mousavi [37] considered the bond–slip effect by changing the yield stress and elastic modulus of the steel bar, and obtained a modified steel bar model. The results revealed the significant effect of bond–slip on total behavior of the member.

Previous studies have shown that most of the proposed bond–slip relationships were derived from the pull-out tests. These bond models, which vary with concrete types and reinforcing steel parameters, are inconsistent with each other. Moreover, the initial stiffness of the aforementioned bond stress–slip curves is infinite, which is impractical and could cause problems in numerical analysis. Overall, using a pull-out test, the local bond stress–slip relationship can be obtained. However, this test does not reflect the change in bond stress along the longitudinal axis of the embedded steel bar in the actual cracking zone. In fact, the local bond stress–slip constitutive relationship changes along the length of the embedded steel bar. For different concretes, the existing literature has proposed position functions that reflect this change [38,39,40]. However, there is still room for research on the distribution of the bond stress between reinforcing steel and concrete along the length of the embedded steel bar. Considering this, this study used an axial tension test to simulate the behavior of the beam member after cracking. In this study, strain gauges were installed at different positions inside the steel bar of the test specimens to measure the changes in the stress of the steel bar along the embedded length, and the relative displacement of the concrete and the steel bar at the crack was measured by linear variable differential transformers (LVDTs). By deducing the changes in stress and strain, the bond stress and slip of the steel and concrete inside the specimen, the position function was derived to reflect these changes, thus achieving a more accurate description of the bond stress–slip behavior.

## 2. Experimental Details

### 2.1. Materials and Mix Proportions

The materials used to make the pull-out specimens included cement, silica fume, fine and coarse aggregates, superplasticizers, and steel bars. The cement used was local ordinary Portland cement conforming to ASTM C150/C150M [41], with a specific gravity of 3.15 and a fineness of 3400 cm^2^/g. The silica fume used was purchased from abroad, and its specific gravity was 2.08. A well-graded aggregate and washed natural sand were selected in accordance with ASTM C33/C33M [42]. Among them, the fine aggregate was natural river sand, and the coarse aggregate was crushed stone with a maximum particle size of 19 mm. The physical properties of these aggregates are listed in Table 3. To ensure that the concrete had good workability, two different superplasticizers produced by Taiwan Jong Shin Company were selected. Among them, HICON HPC 1000 was used for the medium strength concrete, and HICON MTP A40 was used for the high strength concrete. Their basic properties are shown in Table 4. The reinforcement was #8 rebar with a diameter of 25 mm, a cross-section area of 5.07 cm^2^, a rib distance of 30.4 mm, a rib width of 3.7 mm, a rib height of 1.7 mm, and an elastic modulus of 205 GPa.

To study the effect of concrete strength on bonding performance, the concrete was given three designed compressive strengths over 28 days: 20, 40, and 60 MPa. The concrete mix proportions are given in Table 5. In the concrete mix number, the letter C indicates the type of concrete (ordinary concrete), and the number indicates the strength of the concrete (20, 40, or 60 MPa). All the aggregates were treated in a room until the required saturated surface dry condition was reached. The aggregates were then maintained in a room in which the ambient temperature and relative humidity (RH) were controlled at 25 ± 3 °C and 50 ± 5% to prevent moisture changes. When mixing, the cement (silica fume), fine aggregate, and coarse aggregate were first poured into the mixing drum of the mixing machine and mixed thoroughly. Then, water and superplasticizer were poured into the mixing drum and mixed until the concrete slurry was homogeneous.

### 2.2. Proportions Casting of Specimens

**Figure 2:**
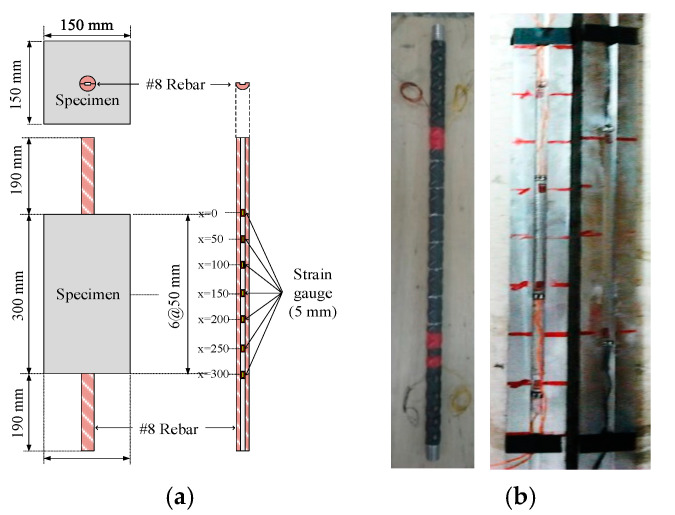
Details of the tension–pull specimen: (**a**) dimensions and cross-section and (**b**) machined #8 rebar.

The details of the specimens for the axial tension test are shown in Figure 2. The cross-section of the tension–pull specimens was a square of 150 mm × 150 mm. The embedded length of the #8 steel bar was 300 mm. On the one hand, it could ensure that the crack spacing was long enough to facilitate the satisfactory variation of the stress in #8 steel bar. On the other hand, the length was short enough to avoid the formation of a transverse crack in the concrete. The #8 rebar was buried in the center of the concrete, and the length of the rebar protruding at both ends was 190 mm. In this study, in reference to the literature [43,44], the #8 rebar was cut into two equal parts along the diameter to obtain the actual steel stress during the test, as shown in Figure 2. Each sawn half-bar was milled to provide a longitudinal groove 4 mm wide and 2 mm deep. In the groove, we installed a 5-mm long strain gauge with a 50-mm interval along the length of the specimen. A total of seven strain gauges were pasted in two small grooves opposite each other along the centerline of the rebar. The upper and lower parts of the rebar were spot-welded together before being embedded in the concrete specimen. Moreover, appropriate threads were prepared at both ends of the reinforcing bar to take a nut in order to secure the two halves tightly together.

Steel molds were used to cast all the tension–pull specimens. The freshly mixed concrete was slowly poured into the tension–pull specimen mold, and a concrete vibrator was used to compact the concrete. For each concrete mix, two tension–pull specimens were cast, for a total of six. For each concrete mixture, six 100 mm diameter × 200 mm high cylindrical specimens, referred to hereafter as the control cylinders, were also cast for the compressive strength test. Moreover, for the split strength test, six cylindrical specimens of each concrete mixture were cast with a diameter and a height of 150 mm and 300 mm, respectively. After the specimens were cast, they were covered with wet linen cloth and polyethylene sheet for 24 h, and then demolded. After the specimens were demolded, they were immediately placed in a laboratory water container for 27 days. Testing was performed 28 days after casting.

### 2.3. Instrumentation and Test Procedures

**Figure 3:**
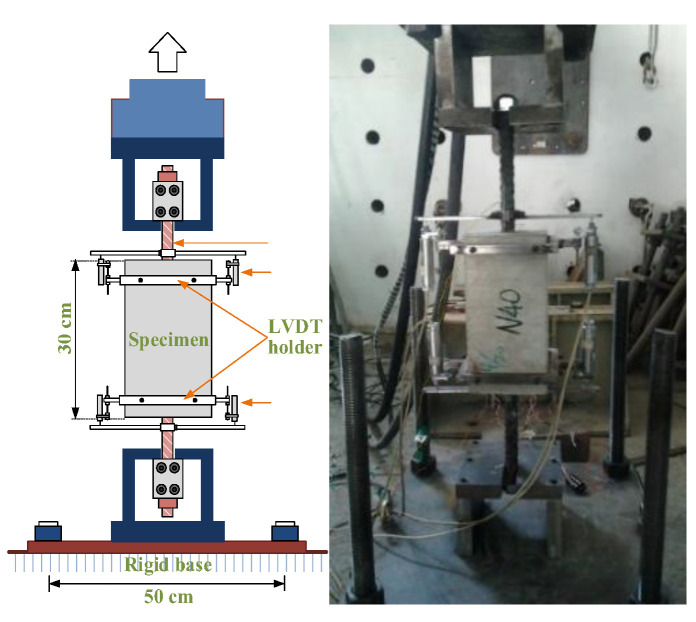
Setup of uniaxial tensile test.

In this study, a machine controlled by a-500 kN MTS servo valve and a specially fabricated testing stand were used to perform the uniaxial tensile testing of the tensile specimens. The schematic diagram of the test setup and the relevant details of the specimens are shown in Figure 3. It can be seen from Figure 3 that the relative bond slip between the #8 rebar and the surrounding concrete was measured by a pair of linear variable differential transformers (LVDTs) installed on both sides of the #8 rebar near the embedded part of the specimen. Under the displacement control, the pulling force was applied at a constant rate of 0.01 mm/s until the designed load was reached. Loading was applied monotonically to the tension–pull specimens in increments of 10 kN from 0 to 180 kN. During the test, the pull-out force was measured by a dynamometer installed in the testing machine. Furthermore, the test progress was monitored on a computer screen. In addition to observing each load increment and displacement data, these data were also stored in the hard disk through a data logger.

### 2.4. Analytical of Measurements

**Figure 4:**
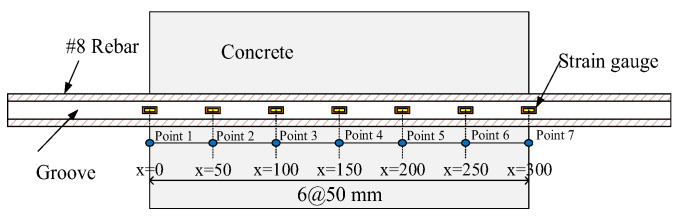
Calculation points for the steel strain.

**Figure 5:**
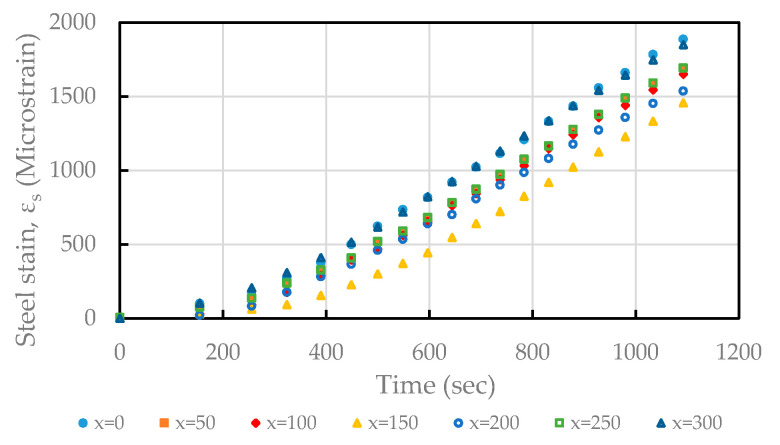
Steel strain and its changing process at various positions inside the tensile specimen during loading (taking concrete C20 as an example).

In the axial tensile test, an axial tensile force was applied to the protruding steel bars at both ends of the specimen. As the load increases, the distribution of tensile stress in the steel and concrete in the specimen also changes. The distribution of the tensile stress is very similar to the distribution of the tensile zone of a general RC flexible member. Therefore, it is generally agreed that the bond–slip relationship obtained from the analysis through the axial tensile test will be closer to the actual situation than the bond–slip relationship obtained from the pull-out test. In the tensile test specimen, the strain gauges installed at different positions inside the rebar are shown in Figure 4. Taking concrete C20 as an example, the actual measured steel bar strain at each calculation point over the course of the test, and its transformation process, are shown in Figure 5. Assuming that the material has linear elastic behaviour, the stress–strain distribution of the steel bar, the stress–strain distribution of the concrete, and the bond stress–slip of the test specimen can be obtained through theoretical analysis methods. The detailed analysis method is as follows.

#### 2.4.1. Definition of the Model

**Figure 6:**
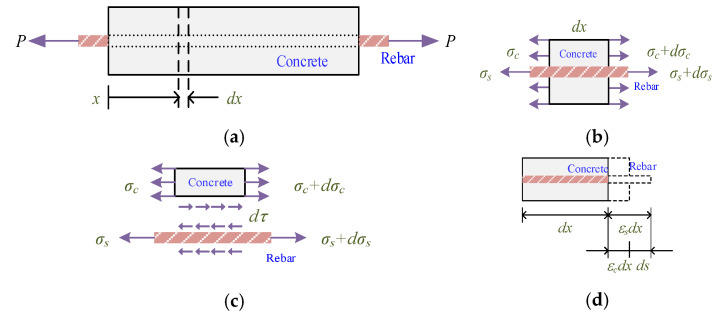
Analytical model: (**a**) diagram of tension–pull specimen, (**b**) equilibrium of prism, (**c**) equilibrium at interface, and (**d**) compatibility of deformations.

The concrete prism analysis model used in this study is shown in Figure 6. This model not only considers the constitutive relationship of the constituent materials but also introduces the bond–slip relationship at the interface. This characteristic overcomes the assumption that there is no slip between the concrete and the steel. The analytical formulation that dominates the behavior of the element (a single bar embedded in concrete with an infinitesimal length of *dx*, as shown in Figure 6a with reference to the cross-section at abscissa *x* is summarized below.

#### 2.4.2. Stress and Strain of Steel

As mentioned earlier, to determine the bond stress along the steel bars, strain gauges installed at various positions can be used to measure the strain of the steel (Figure 4). As shown in Figure 4, there are seven calculation points for bond stress, that is, five intermediate points and two boundary points. The five intermediate points are located in the middle of the embedded length, and the two boundary points are located at the edges of the start and end points of the embedded length. During the loading process, the pasted strain gauge can steadily send real-time data to the acquisition system. Assuming that the steel bars inside the test piece are still in the linear elastic range, then the measured steel bar strain $\varepsilon_{s}$ at each position is substituted into the generalized Hooke’s law to obtain the corresponding steel stress at each position, as shown in the following equation:

(11) $$\sigma_{s}\left(x\right)=E_{s}\varepsilon_{s}\left(x\right)$$

where $\varepsilon_{s}$ = steel strain and $E_{s}$ = Young’s modulus of the steel.

#### 2.4.3. Stress and Strain of Concrete

As shown in Figure 6b, by cutting the segment at *x* and taking a free body, we obtain

(12) $$P=\sigma_{c}A_{c}+\sigma_{s}A_{s}$$

where *P* = applied normal force; $\sigma_{c}$ = stress in the concrete; $A_{c}$ = cross-sectional area of the concrete; $\sigma_{s}$ = stress in the steel; and $A_{s}$ = cross-sectional area of the steel. Furthermore, as shown in Figure 6b, the following equilibrium equation must be satisfied:

(13) $$d\sigma_{c}A_{c}+d\sigma_{s}A_{s}=0$$

Using Equation (13), the concrete stress can be calculated by the following equation:

(14) $$\sigma_{c,i+1}=\sigma_{c,i}-\left(A_{s}/A_{c}\right)\left(\sigma_{s,i+1}-\sigma_{s,i}\right)$$

where $\sigma_{c,i}$ = concrete stress at the *i*th section; $\sigma_{c,i+1}$ = concrete stress at the (*i* + 1)th section; $\sigma_{s,i}$ = steel stress in the *i*th section; and $\sigma_{s,i+1}$ = steel stress in the (*i* + 1)th section. Since the concrete at *x* = 0 is not stressed, the boundary condition is $\sigma_{c}\left(0\right)=0$. Assuming that the concrete inside the specimen still has linear elastic behavior, the concrete strain at each position ($\varepsilon_{c}\left(x\right)$) can be obtained from the generalized Hooke’s law and Equation (14), as shown in the following equation:

(15) $$\varepsilon_{c}\left(x\right)=\sigma_{c}\left(x\right)/E_{c}$$

where $\varepsilon_{c}$ = concrete strain, and $E_{c}$ = Young’s modulus of the concrete.

#### 2.4.4. Bond Stress

Using the force equilibrium (Figure 6c), the relative bond stress can be obtained by

(16) $$\sigma_{s,i+1}-\sigma_{s,i}\;\;=\;-d\tau \left(\pi d_{b}dx\right)$$

where dτ = the relative bond stress between two adjacent positions; $d_{b}$ = steel bar diameter. Applying the boundary condition (i.e., at *x* = 0, τ(0)=0) [45], the bond stress in any segment of the prism can be calculated by the following equation [46]:

(17) $$\tau_{i+1}=\tau_{i}+d\tau$$

where $\tau_{i}$ = bond stress at the ith section, and $\tau_{i+1}$ = bond stress at the (*i* + 1)th section.

#### 2.4.5. Slip between Concrete and Steel Bar

The increment of the local slip (ds(x)) within the infinitesimal steel bar length dx at position *x* can be defined as the difference between the steel strain $\varepsilon_{s}\left(x\right)$ and the concrete strain $\varepsilon_{c}\left(x\right)$ (Figure 6d), as shown in the following equation:

(18) $$ds\left(x\right)=-\left[\varepsilon_{s}\left(x\right)-\varepsilon_{c}\left(x\right)\right]dx$$

where ds(x) = relative slip. Applying the boundary condition (at the central point of the specimen, slip = 0), the slip in any segment of the prism can be calculated by the following equation:

(19) $$s_{i+1}=s_{i}+ds$$

where $s_{i}$ *=* slip at the *i*th section, and $s_{i+1}$ = slip at the (*i* + 1)th section.

## 3. Experimental Results and Discussion

### 3.1. Mechanical Properties of Concrete

On the same day as the uniaxial tensile test, each control cylinder was capped and a compression test was performed to use the result as the concrete compressive strength of the tension–pull specimen. The average compressive strength was calculated by taking the average of three specimens. Table 6 shows that the average 28-day compressive strength of each concrete mixture was close to the design value (i.e., 20, 40 and 60 MPa). In addition, Table 6 also shows the average splitting tensile strength and elastic modulus of each concrete mixture.

### 3.2. Steel Strain Distribution

**Figure 7:**
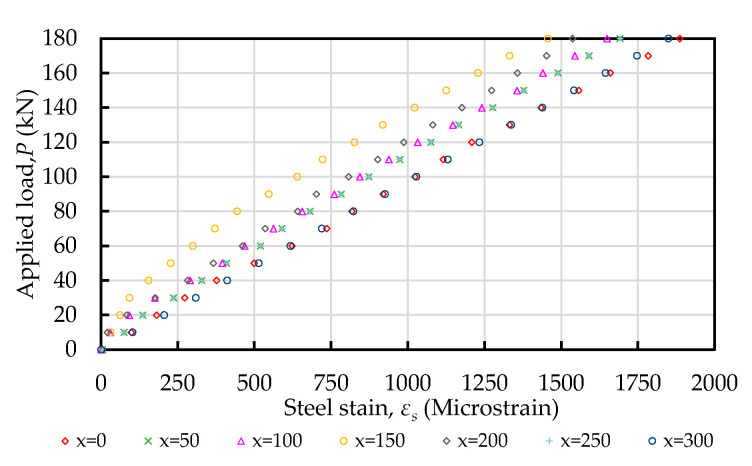
Applied load and steel strain relationship (taking concrete C20 as an example).

**Figure 8:**
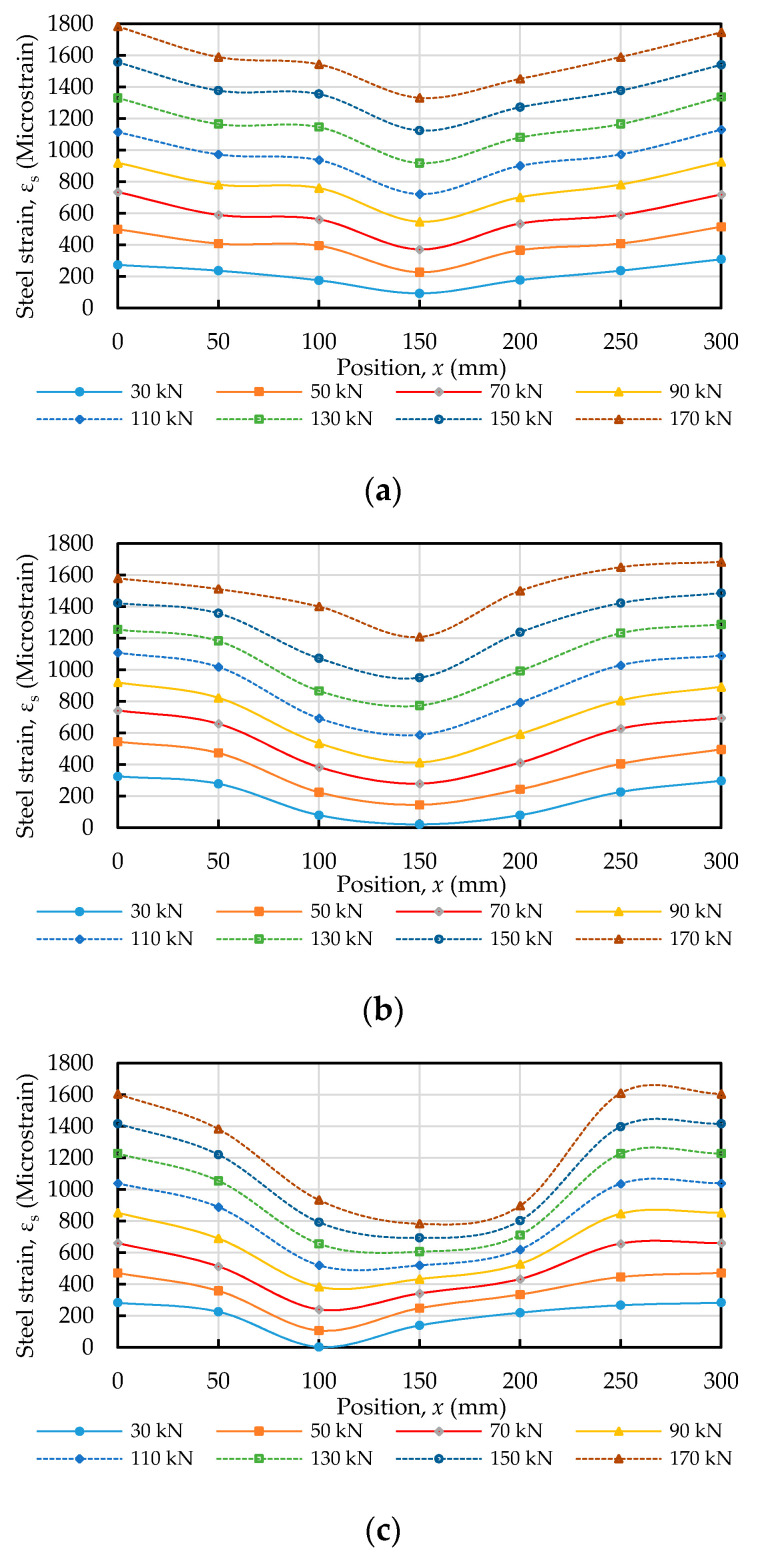
Strain distribution in steels bar subjected to tensile load: (**a**) C20, (**b**) C40, and (**c**) C60.

In the tension–pull specimens, the steel strain was measured using strain gauges installed at various planned positions, as shown in Figure 4. Taking concrete C20 as an example, the applied load and steel strain relationship is plotted in Figure 7. It can be seen from Figure 7 that the steel strain in the specimen increased proportionally with the increase in the load. According to the different concrete strength of the specimens, the steel strain distribution along the longitudinal axis of the steel bars actually measured during the test is shown in Figure 8. It can be seen from Figure 8 that the steel strains in the specimen increased with an increasing load and varied with the position of the strain gauge. As the position of the strain gauge moved from the two ends of the test piece (i.e., *x* = 0 and *x* = 300) to the center, the strain of the steel bar decreased, and the minimum strain value was at the center (*x* = 150). In addition, the strain values that were symmetrical to the center were similar (e.g., *x* = 0 and *x* = 300; *x* = 50 and *x* = 250; *x* = 100 and *x* = 200), which was in line with the characteristics of symmetrical stress. At both ends of the specimen (*x* = 0, *x* = 300), the steel bar strain value was almost equal to the steel bar tensile strain value under the same load. Moreover, it can be seen from Figure 8 that with an increase in the compressive strength of the concrete, the strain of the steel bars in the center and the two sides of the center of the test piece showed a significant decreasing trend. From this point of view, in the vicinity of the center of the specimen, the concrete’s axial tensile force was greater than that near the two sides. When the specimen was subjected to axial tensile force, the concrete near the steel bar was also subjected to tensile force due to the bond effect. When the bond performance is good, concrete can withstand greater tensile stress. Therefore, as the compressive strength of our concrete increased, the strain development of the steel bar near the center of the specimen became smaller.

### 3.3. Concrete Strain Distribution

**Figure 9:**
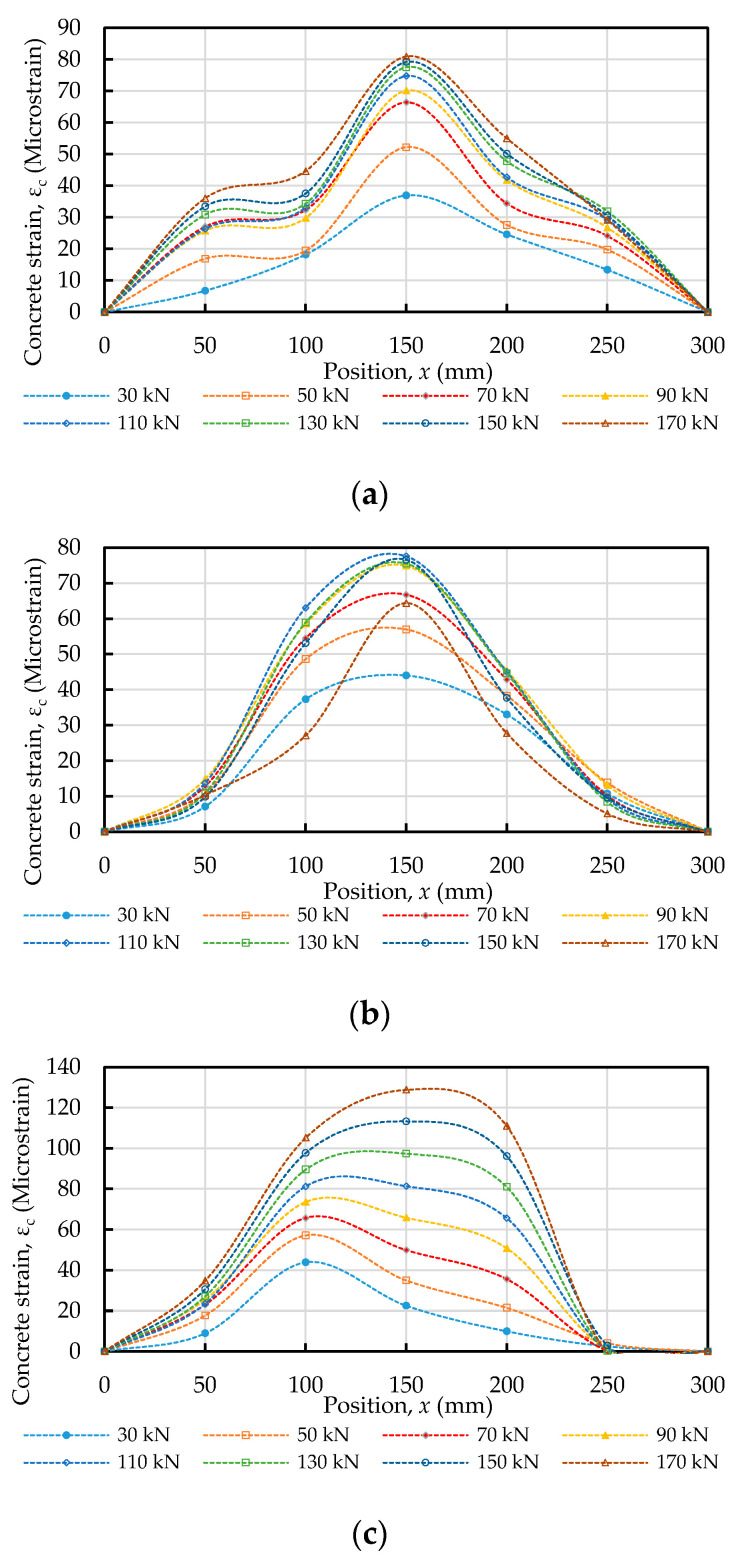
Strain distribution in the concrete subjected to a tensile load: (**a**) C20, (**b**) C40, and (**c**) C60.

The axial stress and strain of the concrete were calculated by Equations (14) and (15), respectively. The calculation results of the concrete strain of the specimen under different axial tensile forces are shown in Figure 9. It can be clearly seen from Figure 9 that in the specimen subjected to any fixed load, the maximum value of concrete strain occurred around the center, and its value increased as the compressive strength of the concrete increased. Moreover, comparing Figure 9 with Figure 8, it can be seen that the concrete strain was less than the steel strain along the overall embedded length.

### 3.4. Bond Stress Distribution

**Figure 10:**
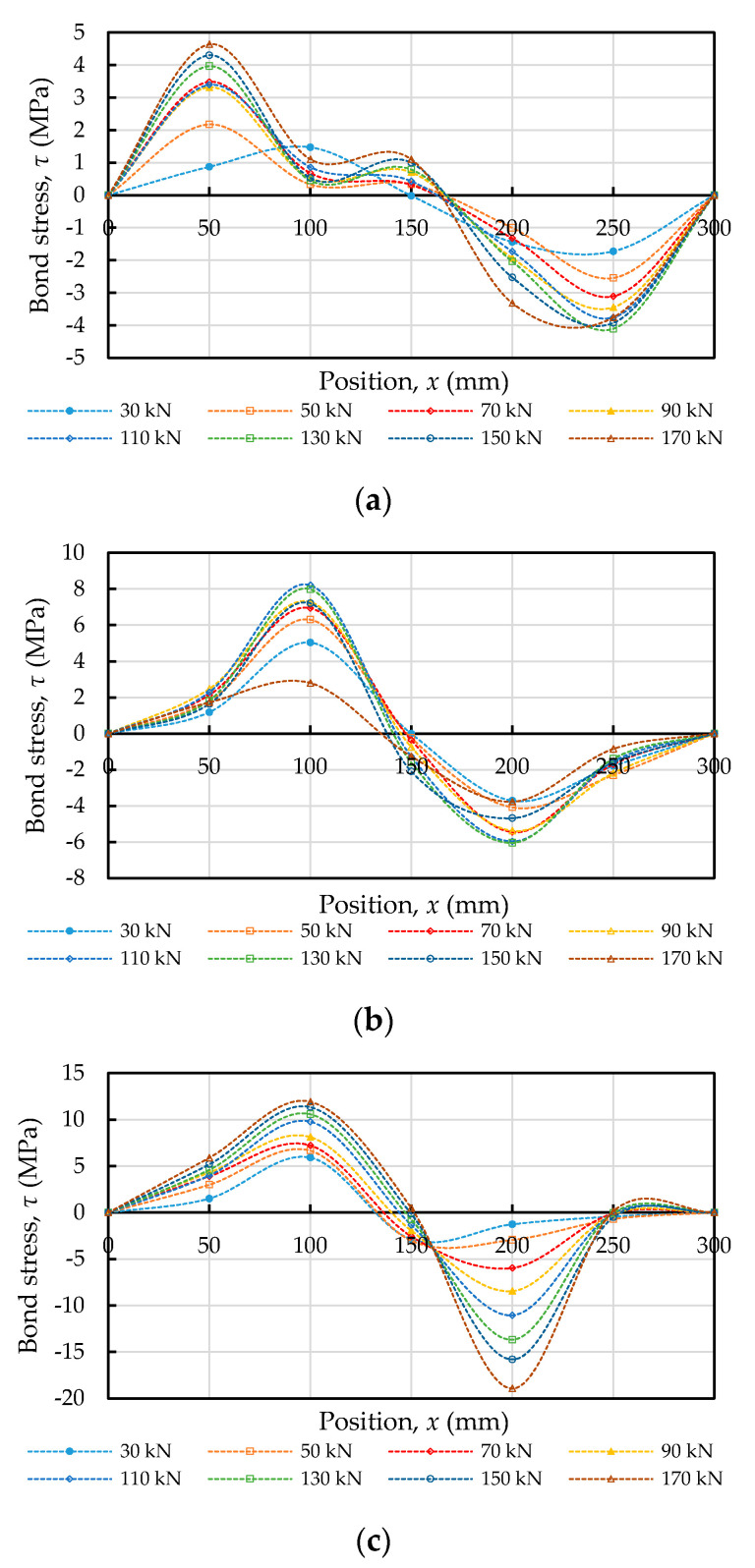
Bond stress distribution along steel bars subjected to tensile load: (**a**) C20, (**b**) C40, and (**c**) C60.

It can be seen from the schematic diagram of the force balance of the steel bar in the lengths of two adjacent positions in Figure 6c that the internal bond stress of the specimen is mainly caused by a change in the steel stress. The bond stress developed by the steel bar in any segment of the prism specimen was calculated by Equation (17), and the boundary conditions were set as *τ*(0) = 0 and *τ*(300) = 0. Figure 10 shows the bond stress distribution curve with respect to several increments of the static tensile load within the service range. As the tensile load increased, the bond stress of almost all measuring points also continued to increase, as shown in Figure 10. The bond stress distribution of the specimen was uneven along the embedded length. Previous studies on the distribution of bond stress in concrete have shown that the assumption of a uniform bond stress is only applicable to short-embedded specimens [47]. In addition, it can be seen from Figure 10 that the change of the bond stress distribution was a sinusoidal waveform; the maximum value of each curve mainly occurred at or near the central anchored point, whereas the minimum value occurred at the anchored midpoint or the loaded end due to the symmetry.

### 3.5. Slip Distribution

**Figure 11:**
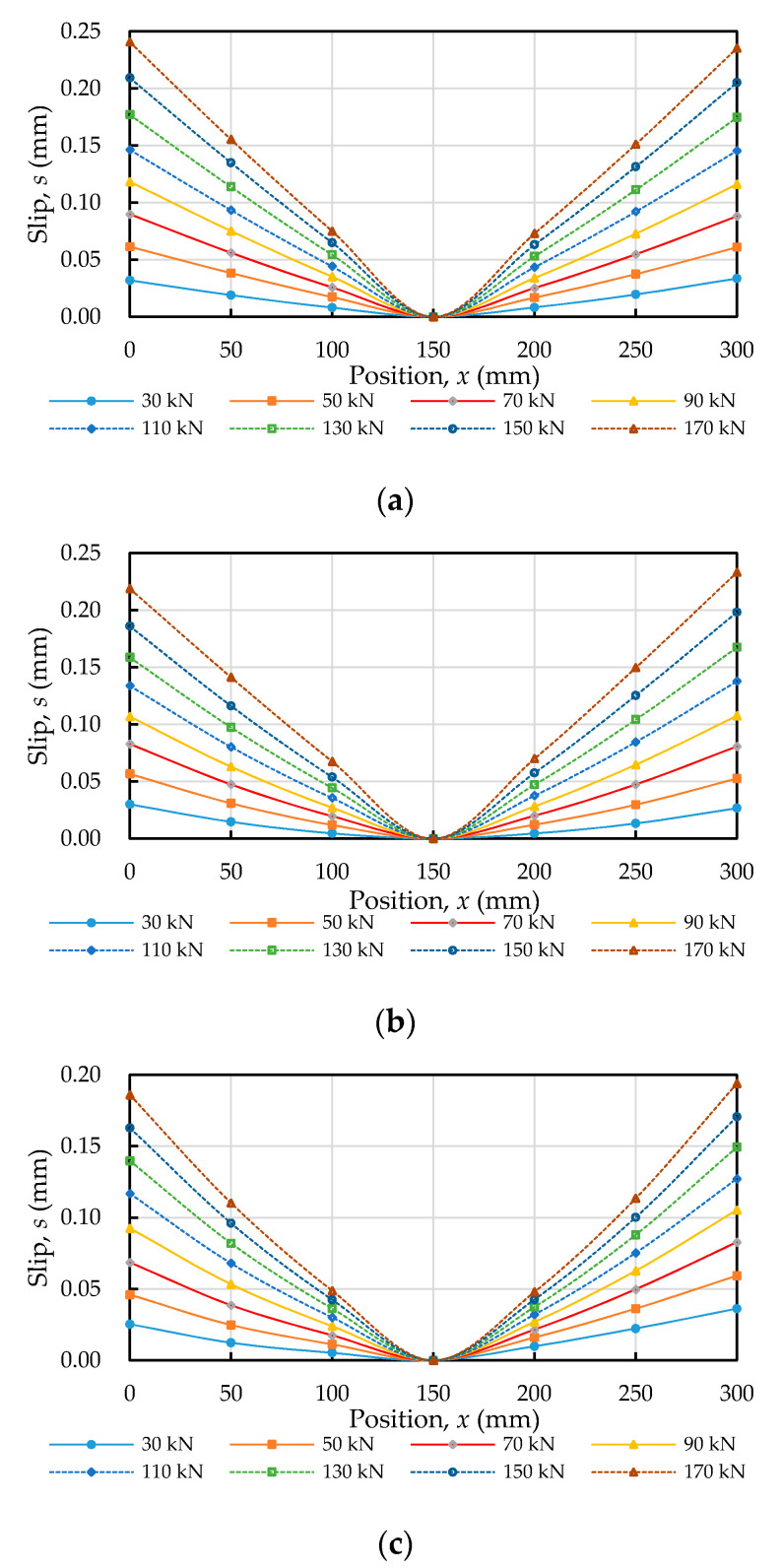
Slip distribution along steel bars subjected to a tensile load: (**a**) C20, (**b**) C40, and (**c**) C60.

According to the diagram showing the compatibility of deformations in the specimen (Figure 6c), the relative slip of the steel bar between two adjacent positions, *ds*(*x*), can be obtained by using Equations (18) and (19). The calculated amount of slip for each specimen subjected to different axial tensile forces is plotted in Figure 11. As shown by the slip distribution curve in Figure 11, in the specimen subjected to any fixed load, the magnitude of the slip varied from zero at the anchored midpoint to a maximum at the ends due to symmetry. In addition, with an increase in the concrete’s compressive strength, the magnitude of the slip distribution curves at each position of the specimen tended to be smaller.

### 3.6. Bond Stress–Slip Relationship

**Figure 12:**
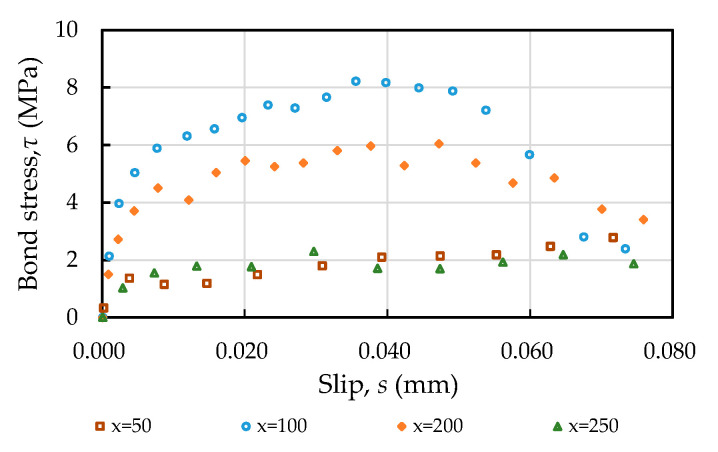
Bond stress–slip relationship curve at different positions of the specimen (taking concrete C20 as an example).

**Figure 13:**
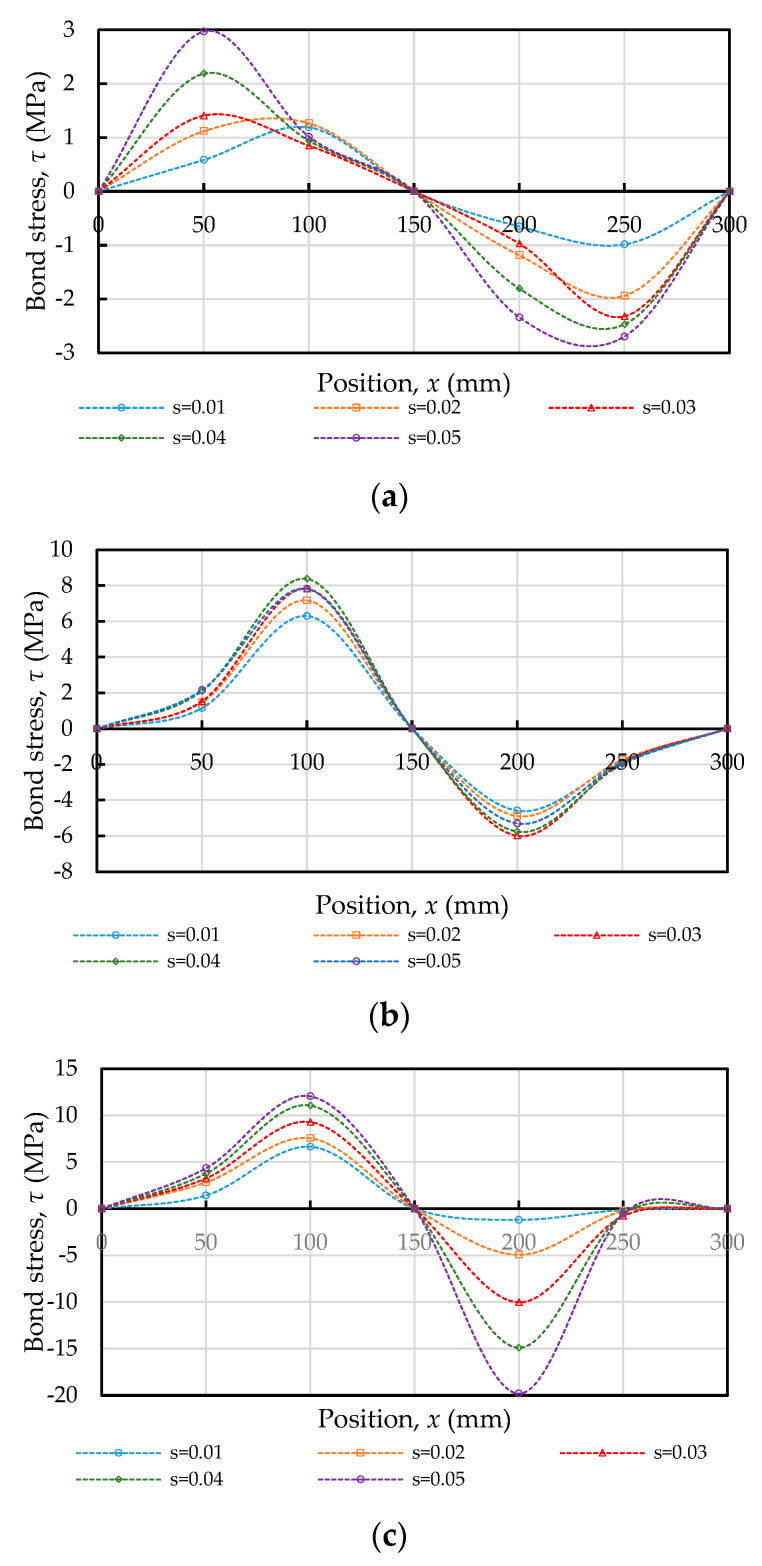
Bond stress vs. slip relationship along embedded length with the same slip value: (**a**) C20, (**b**) C40, and (**c**) C60.

Taking the C40 specimen as an example, under the condition that the axial force is loaded to 100 kN, the bond stress–slip relationship curves of the different positions of the specimen were obtained, as shown in Figure 12. It can be clearly seen from Figure 12 that the relationship between the bond stress and slip at the steel–concrete interface was not consistent but varied with the measurement position. In other words, the bond stress–slip relationship varied with the position of the steel bar; the closer the stress was to the center of the specimen, the curve steeper the curve became and the more the bond stiffness increased. On the contrary, the bond stiffness at the center and both ends of the test piece was zero. In other words, the bond strength and stiffness approached zero at the loaded end or near the central anchored point of the specimen. In addition to measuring the bond stress at different positions under the same slip value, they were also connected with a smooth curve, as shown in Figure 13. Overall, the maximum value of the bond stiffness of the specimen occurred in the vicinity of *x* = 0.3$l_{a}$ − 0.7$l_{a}$ (*x* = distance from the loaded end; $l_{a}$ = half the length of the specimen).

### 3.7. Position Function

**Figure 14:**
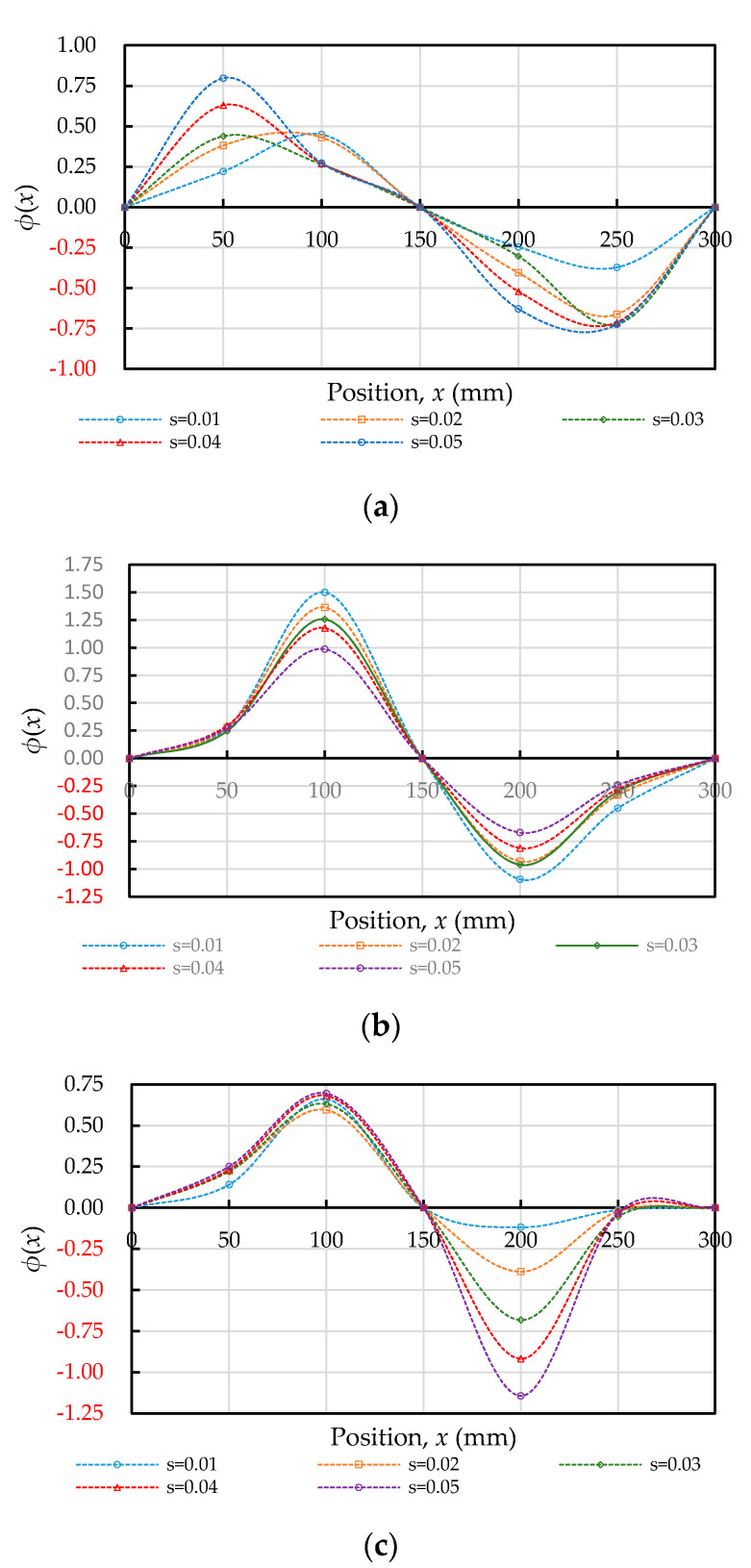
Position function: (**a**) C20, (**b**) C40, and (**c**) C60.

From the previous analysis, it can be known that the bond stress–slip relationship varied along the longitudinal axis of the steel bar. To describe this change, the local bond–stress relationship was obtained by a general local pull-out test [48]. Then, we determined a position function ϕ(x) and expressed the bond stress–slip relationship at different embedded lengths *x* of the steel bar by the product of the two. The ϕ(x) curve of each specimen was drawn, as shown in Figure 14. Clearly, the shape of the position function of the specimen is only related to the relative embedded position, and the curve distribution under different slip values is basically similar. Moreover, comparing the ϕ(x) of each specimen with its bond stiffness curve, it can be clearly seen that the curve shapes of the two are very consistent.

**Figure 15:**
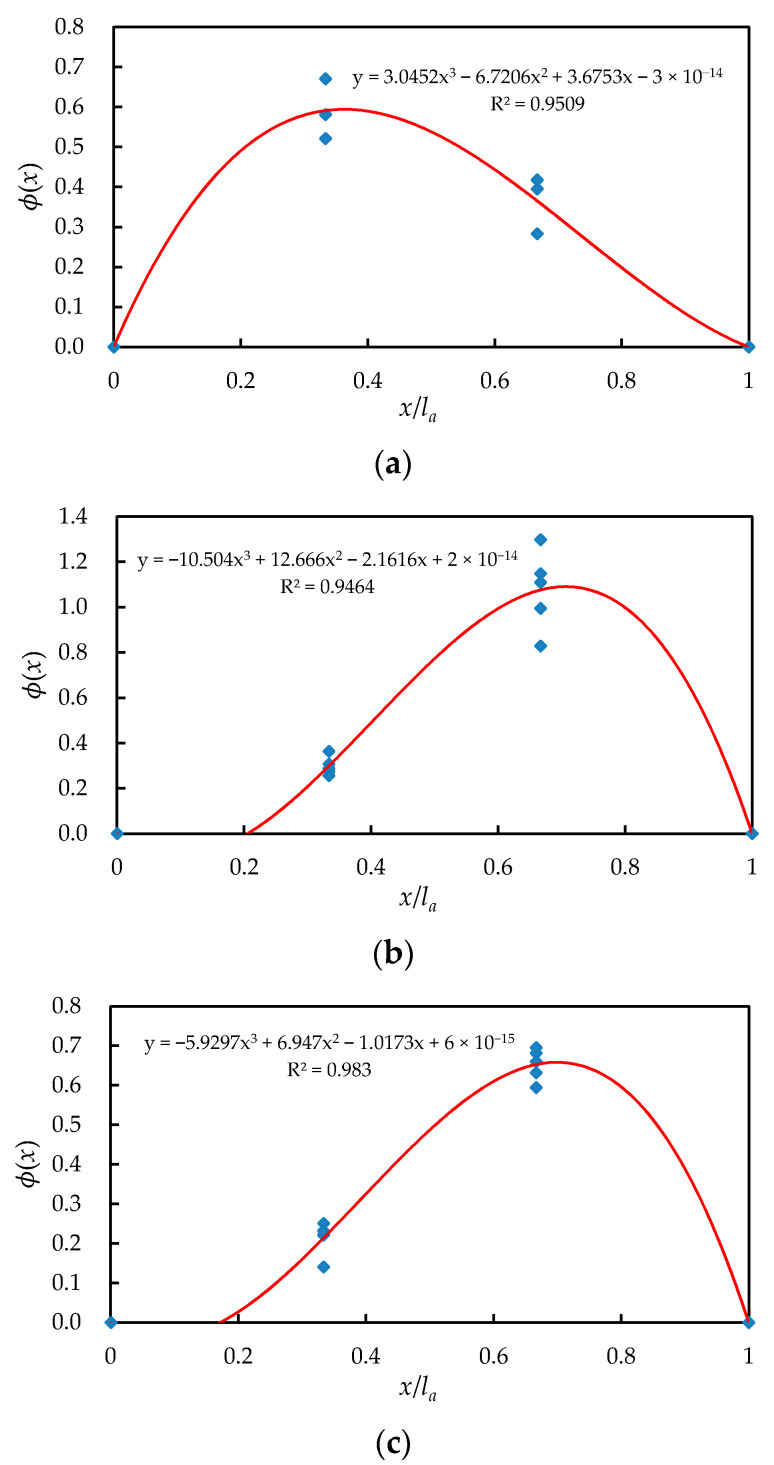
Position function fitting: (**a**) C20, (**b**) C40, and (**c**) C60.

The position function ϕ(x) describes the bond stiffness at different positions and is a relative function. In other words, the shape of the position function ϕ(x) is used to describe the relative magnitude of the bond strength at different positions along the steel bar. Taking the ratio $\left(x/l_{a}\right)$ of the distance from the endpoint of the specimen (*x*) to the half-length of the specimen $\left(l_{a}\right)$ as the parameter, the position function ϕ(x) and its determination coefficient were obtained by regression analysis, as shown in Figure 15. According to the concrete strength, the polynomials of the position function of each specimen are listed as Equations (20)–(22):

(20) $$N20\text{:}\;\phi \left(x\right)=3.05{\left(\frac{x}{l_{a}}\right)}^{3}-6.72{\left(\frac{x}{l_{a}}\right)}^{2}+3.68\left(\frac{x}{l_{a}}\right)-3\times 10^{-14}\;(R^{2}=\;0.95)$$

(21) $$N40\text{:}\;\phi \left(x\right)=-10.50{\left(\frac{x}{l_{a}}\right)}^{3}-12.67{\left(\frac{x}{l_{a}}\right)}^{2}-2.16\left(\frac{x}{l_{a}}\right)+2\times 10^{-14}\;(R^{2}=\;0.95)$$

(22) $$N60\text{:}\;\phi \left(x\right)=-5.93{\left(\frac{x}{l_{a}}\right)}^{3}+6.95{\left(\frac{x}{l_{a}}\right)}^{2}-1.02\left(\frac{x}{l_{a}}\right)+6\times 10^{-15}(R^{2}=\;0.98)$$

### 3.8. Bond Stress–Slip Constitutive Relationship

We can determine the local bond stress–slip constitutive relationship τ(s) using a general local bond pull-out test. The position function ϕ(x) can also be determined as previously stated. Therefore, the bond stress–slip constitutive relationship τ(s,x) can be expressed as follows:

(23) τ(s,x)=τ(s)·ϕ(x)

According to the above equation, the bond stress at different positions along the steel bar can be calculated by the uniaxial tensile test for a specific slip. In addition, the analytical results indicate that the proposed bond stress–slip constitutive relationship is accurate in describing the true bond stress–slip relationship. In other words, it has practical value.

## 4. Conclusions

Based on the analysis model proposed in this paper, the analytical expressions for the stress of the steel bar, the bond stress between the steel bar and the concrete, and the relative slip are derived, and verified by the uniaxial tensile test data. In addition, the change rule of bond stress–slip relationship with different positions is also introduced, namely position function. The analytical results indicate that the proposed bond stress–slip constitutive relationship is very accurate in describing the true bond stress–slip relationship.

## Figures and Tables

**Table 1:** The parameter values for the prediction models for the bond stress–slip relationship.

Parameter	Model Code 2010 [19]	Huang et al. [20]	Harajli et al. [21]
	Confined Concrete	Normal Strength Concrete	Concrete
$s_{1}$	1.0 mm	1.0 mm	0.15 Distance bet. ribs
$s_{2}$	3.0 mm	3.0 mm	0.35 Distance bet. ribs
$s_{3}$	Distance bet. ribs	Distance bet. ribs	Distance bet. ribs
*α*	0.4	0.4	0.3
$\tau_{u}$	$2.5\sqrt{f_{c}^{\prime }}$	$0.4f_{cm}$	$2.57\sqrt{f_{c}^{\prime }}$
$\tau_{f}$	0.4$\tau_{u}$	0.4$\tau_{u}$	$0.9\sqrt{f_{c}^{\prime }}$

**Table 2:** General results for the maximum “local” bond strength in pull-out tests.

Author	Concrete Type	Concrete Strength (MPa)	Embedment Length	Maximum Bond Strength (MPa)
Viwathanatepa et al. (1979) [23]	Normal concrete	30	-	15 ($2.7\sqrt{f_{c}^{\prime }}$)
Hawkins et al. (1982) [24]	Normal concrete	45	2*d_b_*	34 ($5.0\sqrt{f_{c}^{\prime }}$)
Vos and Reinhardt (1982) [25]	Normal concrete	20	3*d_b_*	8 ($1.7\sqrt{f_{c}^{\prime }}$)
		35		17 ($2.9\sqrt{f_{c}^{\prime }}$)
		45		25 ($3.7\sqrt{f_{c}^{\prime }}$)
Eligehausen et al. (1983) [7]	Normal concrete	30	5*d_b_*	14 ($2.6\sqrt{f_{c}^{\prime }}$)
		55		19 ($2.6\sqrt{f_{c}^{\prime }}$)
Fang et al. (2006) [26]	Normal concrete	22–43	4*d_b_*	22 ($2.9\sqrt{f_{c}^{\prime }}$)
Kivell et al. (2011) [27]	Normal concrete	65	4*d_b_*	32 ($4\sqrt{f_{c}^{\prime }}$)
Araujo et al. (2013) [28]	Fiber concrete	60	5*d_b_*	20 ($2.6\sqrt{f_{c}^{\prime }}$)
Choi et al. (2015) [29]	High performance concrete	40	4*d_b_*	35.9 ($5.7\sqrt{f_{c}^{\prime }}$)
		80		37.1 ($4.1\sqrt{f_{c}^{\prime }}$)
		100		35.3 ($3.5\sqrt{f_{c}^{\prime }}$)
		120		36.4 ($3.3\sqrt{f_{c}^{\prime }}$)
Pishro and Feng (2017) [30]	Ultra high performance concrete	82.6	2*d_b_*	17.7 ($1.9\sqrt{f_{c}^{\prime }}$)
		93.6		19.2 ($2.0\sqrt{f_{c}^{\prime }}$)
		107.6		25.1 ($2.4\sqrt{f_{c}^{\prime }}$)
		113.6		27.0 ($2.5\sqrt{f_{c}^{\prime }}$)
Chu and Kwan (2019) [2]	Fiber concrete	51.6–61.3	4.2*d_b_*	19.4–27.1 ($3.1\sqrt{f_{c}^{\prime }}$)

**Table 3:** Physical properties of the aggregates.

Aggregate Type	Specific Weight (SSD)	Water Absorption (SSD) (%)	Unit Weight (Dry-Rodded) (kg/m^3^)	FM
Coarse aggregate	2.63	1.24	1532	-
Fine aggregate	2.56	1.33	-	2.75

Notes: SSD, saturated surface dry condition; FM, Fineness modulus.

**Table 4:** Basic properties of the superplasticizer.

Type	Specific Weight	pH Value	Solid Composition (%)
HPC 1000	1.20	7 ± 1	3.37
MTP A40	1.13	7 ± 1	-

**Table 5:** Concrete mix proportions.

Mix No.	Water/Cement Ratio (W/C)	Cement (kg/m^3^)	Water (kg/m^3^)	Aggregate (kg/m^3^)		SP (kg/m^3^)	Dry Unit Weight (kg/m^3^)
				FA	CA		
C20	0.76	267	203	772	1054	0	2147
C40	0.52	390	203	670	1054	0.78	2194
C60	0.32	591	189	523	1063	6.50	2301

Note: FA, fine aggregate; CA, coarse aggregate; SP, superplasticizer (HICON HPC 1000 for C40 and HICON MTP A40 for C60).

**Table 6:** Mechanical properties of the concrete.

Mix No.	Compressive Strength (MPa)	Splitting Strength (MPa)	Elastic Modulus (GPa)
C20	20.20	2.40	23.32
C40	40.97	2.91	30.22
C60	59.46	3.23	30.72
